# Krüppel-like factor 7 influences translation and pathways involved in ribosomal biogenesis in breast cancer

**DOI:** 10.1186/s13058-022-01562-8

**Published:** 2022-10-03

**Authors:** Anne-Marie Lüchtenborg, Patrick Metzger, Miguel Cosenza Contreras, Victor Oria, Martin L. Biniossek, Franziska Lindner, Klemens Fröhlich, Ambrus Malyi, Thalia Erbes, Nicole Gensch, Jochen Maurer, Andreas Thomsen, Melanie Boerries, Oliver Schilling, Martin Werner, Peter Bronsert

**Affiliations:** 1 Institute for Surgical Pathology, Medical Center – University of Freiburg, Breisacher Straße 115A, 79106 Freiburg, Germany; 2 Faculty of Medicine, University of Freiburg, Freiburg, Germany; 3 German Cancer Consortium (DKTK) Partner Site Freiburg and Cancer Research Center (DKFZ), Heidelberg, Germany; 4 Institute of Medical Bioinformatics and Systems Medicine, Medical Center - University of Freiburg, Freiburg, Germany; 5 Faculty of Biology, University of Freiburg, Freiburg, Germany; 6 Spemann Graduate School of Biology and Medicine, Faculty of Biology, University of Freiburg, Freiburg, Germany; 7 Biotech Research and Innovation Center (BRIC), University of Copenhagen, Copenhagen, Denmark; 8 Institute of Molecular Medicine and Cell Research, Faculty of Medicine, University of Freiburg, Freiburg, Germany; 9 2Nd Department of Pathology, Semmelweis University, Budapest, Hungary; 10 Department of Obstetrics and Gynecology, Medical Center - University of Freiburg, Freiburg, Germany; 11 Core Facility Signaling Factory, BIOSS Centre for Biological Signaling Studies, University of Freiburg, Freiburg, Germany; 12 Department of Obstetrics and Gynecology, University Hospital Aachen (UKA), Aachen, Germany; 13 Department of Radiation Oncology, Medical Center – University of Freiburg, Freiburg, Germany; 14 Tumorbank Comprehensive Cancer Center Freiburg, Medical Center – University of Freiburg, Freiburg, Germany; 15 Core Facility for Histopathology and Digital Pathology, Medical Center - University of Freiburg, Freiburg, Germany

**Keywords:** Ribosomes, Krüppel-like factor 7, Transcription factor, Breast cancer, Proteomics, Transcriptomics, Nucleoli

## Abstract

**Background:**

Ribosomal biogenesis and ribosomal proteins have attracted attention in the context of tumor biology in recent years. Instead of being mere translational machineries, ribosomes might play an active role in tumor initiation and progression. Despite its importance, regulation of ribosomal biogenesis is still not completely understood.

**Methods:**

Using Gene Set Enrichment Analysis of RNA sequencing and proteomical mass spectrometry data in breast cancer cells expressing Krüppel-like factor 7 (KLF7), we identified processes altered by this transcription factor. In silico analyses of a cohort of breast cancer patients in The Cancer Genome Atlas confirmed our finding. We further verified the role of KLF7 the identified ribosomal processes in in vitro assays of mammary carcinoma cell lines and analyses of breast cancer patients’ tissue slices.

**Results:**

We identified the transcription factor Krüppel-like factor 7 (KLF7) as a regulator of ribosomal biogenesis and translation in breast cancer cells and tissue. Highly significant overlapping processes related to ribosomal biogenesis were identified in proteomics and transcriptomics data and confirmed in patients’ breast cancer RNA Seq data. Further, nucleoli, the sites of ribosomal biogenesis, were morphologically altered and quantitatively increased in KLF7-expressing cells. Pre-rRNA processing was identified as one potential process affected by KLF7. In addition, an increase in global translation independent from proliferation and transcription was observed upon exogenous KLF7 expression in vitro. Importantly, in a cohort of breast cancer patients, KLF7-expression levels correlated with aggressiveness of the intrinsic breast cancer subtype and tumor grading. Moreover, KLF7 correlated with nucleolar characteristics in human breast tumor tissue, indicating a role for KLF7 in ribosomal biogenesis.

**Conclusion:**

In mammary carcinoma, KLF7 is involved in ribosomal biogenesis. Alterations of ribosomal biogenesis has far reaching quantitative and qualitative implications for the proteome of the cancer cells. This might influence the aggressiveness of cancer cells.

**Supplementary Information:**

The online version contains supplementary material available at 10.1186/s13058-022-01562-8.

## Background

Breast cancer is the most prevalent type of cancer, with the highest incidence and leading cause of cancer-related deaths in women worldwide. The most aggressive intrinsic subtype, triple negative breast cancer (TNBC), accounts for 10–20% of all breast cancer diagnoses and has limited treatment options and low 5-year survival rates [1].

Ribosomes have been considered as passive translation machineries for decades, stable in stoichiometry and with limited control over the translation process. In recent years, ribosomes have been identified as flexible components and drivers of cancer initiation and progression, and have been proposed as drug targets [2–5]. One substance targeting the eukaryotic ribosome, homoharringtonine, has already found its way into clinical practice for chronic myeloid leukemia therapy [6].

Ribosomal biogenesis is a highly regulated, multistep process, starting with the transcription of ribosomal DNA followed by association of rRNA with the ribosomal proteins that requires the concerted action of over 200 proteins. Ribosomal biogenesis takes place at the nucleoli, which adapt to the ribosomal need. Nucleolar morphology and quantity are altered in a wide range of malignant tumors and constitute a factor in tumor diagnosis and grading in routine histopathological diagnostics [7, 8].

Alterations in the ribosome imply deviations in the translation of different mRNAs [2–5, 9, 10]. Mutations and deletions in ribosomal proteins or ribosome biogenesis factors were first linked to rare congenital diseases, the ribosomopathies, which are accompanied by a hyperproliferative phase with an increased risk of developing cancer [11]. Lately, changes in ribosomal composition have been directly related to cancer. In breast cancer, changes in expression of the ribosomal proteins RPS9, RPS14, RPL5, RPL10, RPL11, and RPL39 have been related to tumor initiation and progression [12–16]. RPL15 was identified to promote metastasis and translation of regulators of translation and cell cycles in circulating breast tumor cells [17]. Moreover, specific ribosomal mRNA patterns were detected in cell lines and in breast cancer tissue specimens that solely and successfully distinguished a healthy from a pathological condition [18]. Alterations in the ribosome stoichiometry imply deviations in the translation of different mRNAs [4]. Ribosome specialization might reflect an adaptation to prolonged environmental changes such as continued nutrient depletion or hypoxia [19].

The transcription factor Krüppel-like factor 7 (KLF7) has increasingly attracted attention in the context of cancer development [20, 21]. KLF7 is a member of the conserved Klf/Sp1 transcription factor family and is widely expressed in the human body, e.g., in glandular cells of the digestive tract and in lymphoid tissue of the appendix. In most malignant tumors, KLF7 is present at high expression levels (Human Protein Atlas available from www.proteinatlas.org [22]). It has been demonstrated that KLF7 is involved in neuronal differentiation during development, and in negative or positive regulation of proliferation in hematopoietic cells, myoblasts, and preadipocytes, depending on the cell type [23–26]. KLF7 is a target of TP53 and regulates Golgi complex integrity in pancreatic cancer cells [20]. Despite the high abundance of KLF7 in healthy and pathologic tissues, the biological role of KLF7 is still poorly understood.

Here, we investigated the role of KLF7 expression on cellular mechanisms in breast cancer. Using transcriptomics and proteomics approaches in mammary carcinoma cell lines and in patient tissue samples we aimed at identifying and exploring KLF7 regulated processes.

## Materials and methods

### RNA sequencing (RNA-Seq) analyses

Cells were grown in a cell culture dish to 80% confluency, harvested, and RNA extracted using the E.Z.N.A. Total RNA Kit I (Omega Bio-tek) according to the manufacturer’s instructions. Library prep. and sequencing were performed at the Genomics and Proteomics core facility, DKFZ Heidelberg. RNA quality was verified and was above RIN > 9,5 for all samples. Library preparation was started with 500 ng of input. The raw RNA sequencing files were pre-processed with trimmomatic [27] to ensure sufficient read quality by removing adapters and reads in low-quality segment regions with a base quality below 20. Subsequently, the reads were 2-pass aligned using the STAR aligner [28] and the GRCh37 reference genome from Ensembl. Alignment was followed by normalization and differential expression analysis with the R/Bioconductor [29, 30] package DESeq2 [31]. Genes were considered significant with an adjusted p-value (FDR corrected, according to Benjamini-Hochberg) *p* < 0.05.

### Gene set enrichment analysis

Gene set enrichment analysis (GSEA) of signaling pathways was performed as implemented in the R/Bioconductor package GAGE (Generally Applicable Gene-set Enrichment analysis) [32], with signaling pathways from gene ontology (GO) [33, 34], ConsensusPathDB [35, 36], KEGG [37] and Reactome [38]. Pathways were considered significant with an adjusted p-value (Benjamini–Hochberg) < 0.05.

### Explorative proteomics

MDA-MB-231 cells with KLF7 over expression (KLF7OE) or control plasmid were grown to 80% confluency, washed twice with ice cold PBS, and harvested by scraping. Sample preparation was performed as previously described [39, 40]. Briefly, cell pellets were lysed with 0.1% Rapigest, 0.1 M Hepes pH 8.0 supplemented with protease inhibitors, and sonicated for 20 cycles. Protein concentration was determined by BCA assay (Thermo Scientific). Protein (100 µg) was reduced with 5 mM DTT for 15 min at 37 °C and alkylated with 15 mM 2-iodoacetamide for 15 min in the dark. Proteins were tryptically digested with sequencing-grade trypsin in a 1:25 ratio for 2 h at 50 °C, followed by incubation at room temperature for 18 h. Subsequently, Rapigest was degraded by acidification. Peptides were cleared using the iST columns with triethylamine to ensure compatibility with TMT labelling (PreOmics, Martinsried, Germany). Samples were labeled using TMT-11-plex and fractionated by high-pH, reverse phase chromatography (Agilent 1100 HPLC). Dried samples were resolubilized in 0.1% formic acid and analyzed using an Orbitrap Q-Exactive Plus (Thermo, Bremen, Germany). Proteins were identified and quantified in three biological replicates per cell line. Data was analyzed using MaxQuant as described [39, 40].

### TCGA (The Cancer Genome Atlas) analysis

The TCGA data was accessed and downloaded with the TCGAbiolinks R package [41]. For the analyses, the TCGA-BRCA cohort with the Gene Expression Quantification data type as well as corresponding mutation data was used.

After normalizing the data set, the distribution of the *KLF7* gene expression was analyzed. Subsequently, the cohort was divided according to the quantiles of the distribution. For the gene set enrichment analysis, we compared the samples in the top 33% quantile to the samples in the bottom 33% quantile.

Additionally, the expression of *KLF7* was compared between patients with a functional *TP53* mutation to WT patients. Silent mutations are not considered functional and were therefore excluded. The mean expression values of each group were tested for statistical significance (*t*-test).

### Statistical analysis

Statistics for proteomics were calculated using R4.0.3. Cell culture experiments were statistically analyzed using GraphPad Prism 5. Patient data were analysed using SPSS Version 27. Descriptive statistics with median and percentage of total, as well as estimated median survival, were calculated. The p-value for significance was defined < 0.05. For survival analysis, Kaplan Meier method was performed. Correlations between *KLF7* expression (cytoplasmic, nuclear) and clinico-pathological features were calculated using Pearson, Spearman’s rho, and Kendall rank correlation.

### Cell culture

MDA-MB-231 cells were grown in DMEM (Dulbecco’s modified Eagle’s medium)/F12 supplemented with 10% FCS and 1% penicillin/streptomycin (P/S). MCF7 were cultured in DMEM, 10% FCS, and 1% P/S. All cell lines were authenticated using Multiplex Cell Authentication by Multiplexion (Heidelberg, Germany), as recently described [42]. The SNP profiles matched known profiles.

Stable KLF7-expressing MDA-MB-231 cells were generated by viral transduction from the Core Facility in the Signalhaus of the Albert-Ludwigs-University of Freiburg. Stable clones were selected, with puromycin and positive cells sorted according to fluorescent intensities. Stable KLF7-expressing MCF7 cells were generated by nucleofection (Nucleofector 2b, Lonza) followed by selection with 0.9 mg/ml G418.

### qPCR

Total RNA was isolated using the Total RNA Purification Kit (#PP-210L, JenaBioscience) and cDNA generated using random primer mix (#S1330S, NEB) and Maxima Reverse Transcriptase (#EP0742, ThermoFisher Scientific) according to the manufacturer’s instructions. Real-time qPCR was performed with the PowerUp SYBR Green Master Mix (A25780, Applied Biosystems) on an Applied Biosystems QuantStudio 6-flex real-time PCR System. Primers were as following: KLF7_for AGCTACAACTTGTCCACGA, KLF7_rev ATTCAAGGCATGTCTGCTG, XPO1_for AGCAAAGAATGGCTCAAGAAGT, XPO1_rev TATTCCTTCGCACTGGTTCCT, NXF1_for AAGAGGCGGTTCTGGTATTCG, NXF1_rev TAGGGGTTGTATCGTACTCGG, NXT1_for CTTCCAGCGAGTTCCAAATCA, NXT1_rev CAGATGACAACAAGGACCGTG, NHP2_for CCCCACCTGTGTGATAATGGT, NHP2_rev GCACTCATCGTAAGCCTCCT, NOP10_for CAGTATTACCTCAACGAGCAGG, NOP10_rev GGCTGAGCAGGTCTGTTGTC, XRN1_for TCCAACTGTATCACACCAGGA, XRN1_rev GCTTTGCTTTCTCGGATCTGA, XRN2_for CCTTCGGCTTAATGTTCTTCGT, XRN2_rev TGAAAACCCAGTCATCAATGCT, NVL_for GAATTGTAGCCCAACTCCTAACC, NVL_rev GTCTGGTCGATTAGTAGCTCCA, POP1_for AGAGGTGTAAAGCACCACAGT, POP1_rev GCTGTCGTGAAGTTCCAGG, RMRP_for CGTAGACATTCCCCGCTTCC, RMRP_rev GCGTAACTAGAGGGAGCTGAC. ActB primers for normalization were from Primer design (HK-SY-HU-1200). Fold changes were calculated using the 2^−ΔΔ*C*t^ method.

### Western blot

Cells were harvested and proteins extracted in RIPA buffer (150 mM NaCl, 1% NP-40, 0.5% Sodium deoxycholate, 50 mM Tris–HCl pH 8.0, 2 mM EDTA, 0.1% SDS) including 1× protease inhibitor (Complete Protease inhibitor cocktail, Roche) for 30 min, followed by centrifugation for 15 min (13,000 rpm). For western blotting, 5–10 µg of total protein was loaded on 4–15% Mini-PROTEAN TGX Precast Protein Gels (BioRad) and transferred to PVDF membranes at 100 V for 1 h. Membranes were blocked in 5% milk powder/TBST for 1 h. Primary antibodies were anti-KLF7 (ab197690, Abcam, 1:1000), anti-beta-Tubulin (MA5-16308, ThermoFisher, 1:3000). Secondary antibodies were anti-rabbit-HRP and anti-mouse-HRP (1:25000, JacksonImmunoResearch). Signals were detected using the SuperSignal West Femto Maximum Sensitivity Substrate (ThermoFisher). For reprobing, membranes were stripped with mild stripping buffer (Abcam, 0.2 M glycine, 0.1% SDS, 1% Tween, pH 2.2).

### Proliferation assay

Proliferation was measured using the WST-8-based Rotitest Vital assay (Carl Roth). Briefly, cells were seeded at 5*10^3^ cells/well density in 96 well plates. Every 24 h, 10 µl Rotitest Vital reagent was added to the cells and absorbance was measured at 450 nm in a Tecan M200 plate reader after 1 h of incubation. Medium was used as negative control. Each time point was measured in technical triplicates.

### Cell cycle analysis

For cell cycle analysis, 10^6^ cells were resuspended in 1 ml PBS and 2.5 ml 100% ice-cold ethanol added dropwise under vortexing for fixation. Cells were incubated overnight at 4 °C, washed with PBS, and stained with 50 µg/ml propidium iodide, 0.1% Triton-X100, and 100 mg/ml RNase A for 10 min at 37 °C. Stained cells were measured by flow cytometry (BD Bioscience). 20,000 cells were analyzed per condition. Analysis was performed with Kaluza Analysis 2.1.

### Quantification of transcription and translation

Transcription and translation were quantified using Click chemistry and the CuAAC cell reaction buffer Kit (Jena Bioscience). 5*10^4^ cells were seeded per well in a 96 well optical bottom plate and left to settle overnight. To metabolically label nascent RNA, 1 mM 5-ethynyl uridine (5-EU) in medium was added to the cells and incubated for 60 to 120 min. To measure translation, cells were incubated with 50 µM L-homopropargylglycine (L-HPG, Jena Bioscience) for 45 min. Cells were fixed with 3.7% formaldehyde for 15 min, washed with PBS, 3% BSA, and permeabilized with 0.5% Triton-X100/PBS for 20 min. 5-EU or L-HPG were visualized using the CuAAC Cell Reaction Buffer Kit (Jena Bioscience) according to the manufacturer’s instructions. Briefly, after washing with 3% BSA/PBS, cells were incubated with CLICK reaction cocktail containing 20 µM 5-TAMRA-Azide (Jena Bioscience), 33.33% CuSO4, 166.66 mM THPTA, and 100 mM Na-Ascorbate (Jena Bioscience) for 30 min at room temperature. Cells were washed twice with 3% BSA/PBS and twice with PBS and nuclei stained with DAPI for 2 min. Images were acquired on a AxioVision microscope (Carl Zeiss) using identical settings.

### Patients and cohort

Core needle biopsies of 77 female breast cancer patients, initially diagnosed between 2004 and 2009 at the Department of Obstetrics and Gynecology, University Medical Center Freiburg were included in the study. Written informed consent was obtained from all patients before study inclusion. Ethics approval was obtained by local authorities of the Ethics Committee of the University Medical Center Freiburg (REF.: 523-19, 6.11.2019). To obviate interferences between neo-adjuvant therapeutic interventions and tumor biology, initial core needle biopsies were included. Subsequently, all patients were surgical treated at the Department of Obstetrics and Gynecology, University Medical Center Freiburg. Due to the small amount of tumor tissue within the biopsy, the immunohistologic subtype was adapted [43] without including Ki67 staining.

### KLF7 immunohistochemistry

Formalin fixed, paraffin embedded (FFPE) core needle biopsies were transferred on a tissue microarray (TMA). Microtome sliced in 2 µm sections were deparaffinized and pretreated in 0.1 M citrate buffer, pH 6.0 in a pressure cooker for 2 min for antigen retrieval. Subsequently, slides were washed in wash buffer (DAKO) followed by incubation with the primary antibody (anti-KLF7, HPA030490, SigmaAldrich) and incubation in H_2_O_2_ for 5 min, rabbit linker for 60 min, and horseradish peroxidase and secondary antibody for 20 min. Finally, slides were incubated with 3,3′-dianinobenzidine for 10 min. After hematoxylin counterstaining, slides were mounted in xylene. Immunostaining was evaluated with QuPath 0.2.3 [44]. Tumor regions were annotated with object classifier, and nuclear and cytosolic positive staining quantified using the positive cell detection tool.

### AgNOR staining

Silver staining of nucleoli was performed according to Trerè et al. [45]. Briefly, cells of control and KLF7OE conditions were cultured on µ-slides (IBIDI), fixed with 100% ethanol at − 20 °C for 5 min, post-fixed in Carnoy’s solution (absolute ethanol: glacial acetic acid 3:1), and hydrated through graded alcohols to water. Cells were stained in one volume of pre-warmed 2% gelatin in 1% formic acid and two volumes of 50% silver nitrate solution at 37 °C for 12 min. FFPE TMAs were boiled in 0.1 M citric acid for 25 min in a pressure cooker and rinsed well in water before immersing in staining solution for 25 min. Slides were washed in distilled water, dehydrated, and mounted. Quantification was performed with QuPath 0.2.3 [44]. Nuclei were annotated using the cell detection command, and nucleoli with the subcellular spot detection. For each condition, 5000 to 20,000 cells were analyzed.

## Results

### KLF7 influenced ribosomal pathways in vitro in RNA-Seq

The transcription factor KLF7 was expressed at high levels in physiological conditions in many tissues and prominently present in different tumor types (Human Protein Atlas available from http://www.proteinatlas.org [22]). In the Human Protein Atlas, immunohistologic staining of breast cancer tissue with antibody HPA030490 showed a nuclear KLF7 expression of medium to high intensity in > 70% of all patients and in some breast cancer tissue cytoplasmic staining (data available from v19.3.proteinatlas.org [22]). High KLF7 mRNA levels correlated with significantly worse survival in patients, as demonstrated by the mRNA data of 1063 female breast cancer patients (*p* = 0.045) (data available from v19.3.proteinatlas.org). Here, we aimed to identify the yet unknown downstream targets of KLF7 and pinpoint the molecular function of KLF7 in breast cancer.

**Fig. 1:**
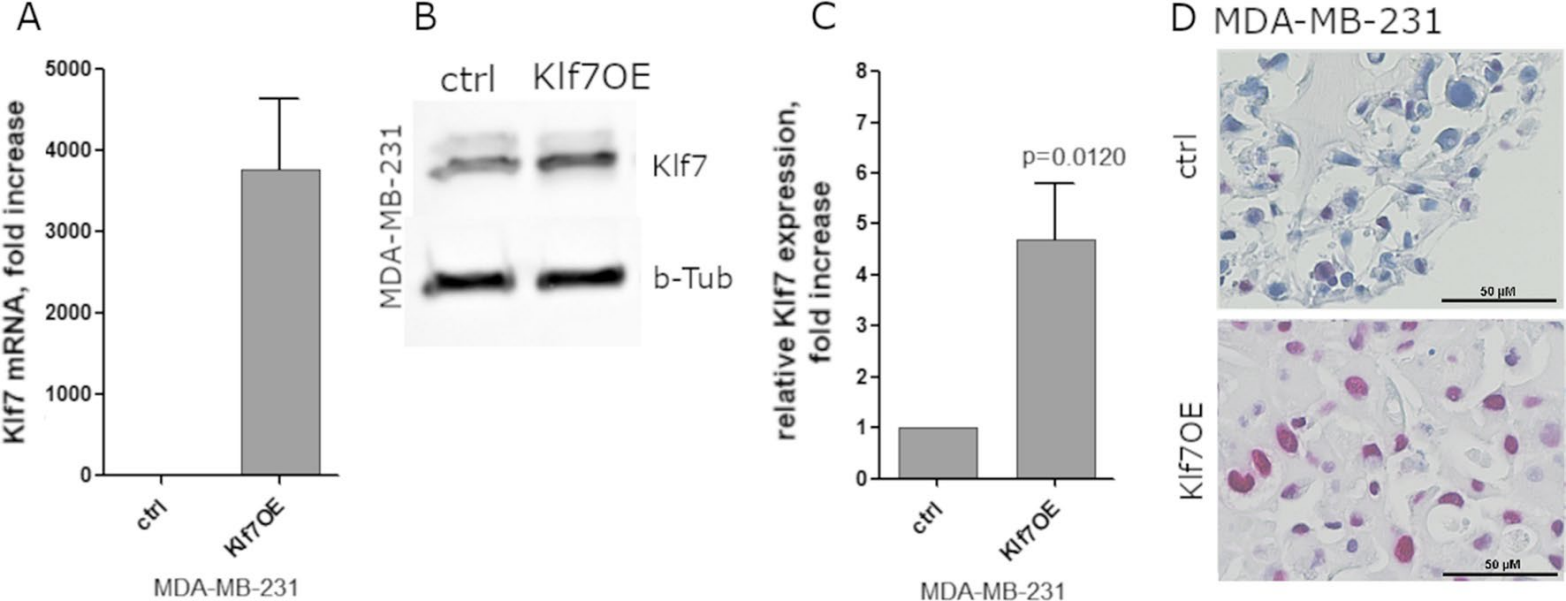
KLF7 overexpression in breast cancer MDA-MB-231 cells. **A** KLF7 mRNA strongly increased upon expression of KLF7. **B** Western blot of MDA-MB-231 control and KLF7OE cells demonstrated increased KLF7 protein levels. **C** Densitometric quantification of western blot against KLF7 in MDA-MB-231 and MCF7 cells, *n* = 4. **D** KLF7 staining in formalin-fixed, paraffin-embedded cell spheres showed nuclear localization of KLF7 in MDA-MB-231

As a transcription factor, KLF7 likely regulates a vast number of mRNAs involved in several pathways. To explore genes regulated by KLF7 and identify KLF7-regulated mechanisms, transcriptomic analysis was performed in mammary carcinoma cells. Therefore, a stable KLF7-overexpressing triple negative, basal-type, mammary carcinoma cell line MDA-MD-231 was generated, which mimics high expression levels in highly aggressive tumor types. Compared to baseline expression level, KLF7 mRNA was induced approximately 3800-fold (Fig. 1A) and KLF7 protein expression was increased by 4.5-fold (Fig. 1B, C). The subcellular KLF7 localization was investigated with anti-KLF7 staining in FFPE (formalin-fixed paraffin embedded) MDA-MB-231 cell spheres, demonstrating a strong nuclear KLF7 expression (Fig. 1D).

RNA-Seq was performed and compared between the MDA-MB-231 KLF7OE and MDA-MB-231 control cells transduced with empty vector. In total, 31,319 RNAs were identified. Of those, 220 were significantly dysregulated in the KLF7OE condition compared to MDA-MB-231 control cells (*p* < 0.05, adjusted p-value according to Benjamini-Hochberg, three biological replicates per cell line).

**Fig. 2:**
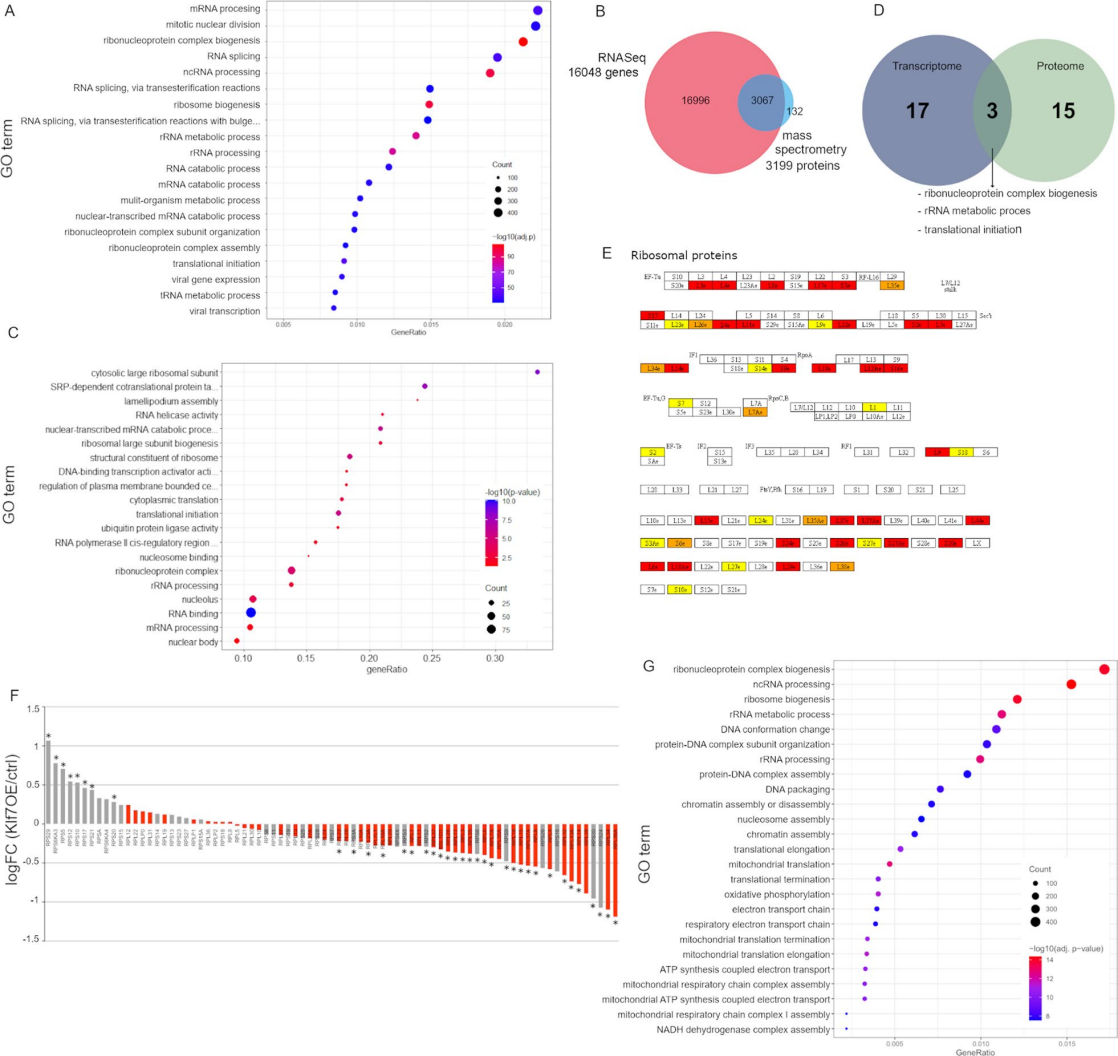
Transcriptomic and proteomic data indicated that KLF7 regulated ribosomal pathways. **A** GSEA of RNA-Seq data showing the top identified GO terms. **B** Overlap of identified genes and proteins in RNA-Seq and mass spectrometry. **C** GO term analysis of shot-gun proteomic data depicted the most regulated pathways. **D** Top significant GO-terms identified in transcriptomics and proteomics data overlapped. **E** Overlapping KEGG pathway of proteomics and transcriptomics data. Red: significantly altered proteins found in mass spectrometry; yellow: significantly modified genes identified in RNA-Seq; orange: compounds found in both screens. **F** Expression levels of ribosomal proteins in MS data in KLF7 compared to control. Red: 40S subunit; grey: 60S subunit. *p < 0.05. **G** Top regulated terms of the gene set enrichment analysis of The Cancer Genome Atlas (TCGA) data in patients stratified according to their KLF7 expression level

KLF7-influenced processes and pathways were identified by a GSEA (gene set enrichment analysis). The analysis results demonstrated highly significant downregulation of several pathways involved in ribosome biogenesis/rRNA processing utilizing the ConsensusDB, Reactome, KEGG, and GO (Gene Ontology) databases. Interestingly, we identified pathways related to ribosomal biogenesis among the top ten downregulated terms in all databases. The most significant pathways were “ribosome biogenesis in eukaryotes” (*p* = 4.3E−30) in the ConsensusDB, “Ribonucleoprotein complex biogenesis” (*p* = 9.71E−111) in GO, and “rRNA processing” (*p* = 1.61E−78) and “hsa03010 Ribosome” (*p* = 4.61E−47) in Reactome and KEGG, respectively (Fig. 2A, demonstrating GO terms). Significantly, upregulated GO and KEGG terms included processes that relate to the extracellular matrix and to the cells matrix attachment [e.g., GO:extracellular matrix (*p* = 2.4E−7) and GO:cell substrate junctions (*p* = 7.3E−7), hsa:04144 Endocytosis (*p* = 1.47E−09), hsa:04512 ECM-receptor interaction (*p* = 1.03E−07)], and hsa:04060 cytokine-cytokine receptor interaction (*p* = 8.86E−07).

### KLF7 influenced ribosomal pathways in vitro in explorative proteomics

It has been demonstrated that transcriptomics and proteomics datasets overlap only partially [46]. Therefore, RNA-Seq findings were corroborated on protein level by explorative proteomics in KLF7OE MDA-MB-231 cells. In total, 3199 proteins were proteomically identified, of which 757 were significantly dysregulated between the two conditions MDA-MB-231 KLF7OE and MDA-MB-231 control cells (adjusted *p* < 0.05, limma-moderated t-test). Of the identified proteins, 3067 were covered by RNA-Seq while 132 proteins were uniquely detected by LC–MS/MS (Fig. 2B). Furthermore, we performed gene ontology analyses to identify regulated pathways on the protein level (Fig. 2C). Gene sets enriched in our proteomics data were attributed to the GO-terms cytosolic large ribosomal subunit (*p* < 0.0001), ribosomal large subunit biogenesis (*p* = 0.00053), structural constituent of ribosome, ribonucleoprotein complex (both *p* < 0.0001), and rRNA processing (*p* = 0.00172).

### Corroboration of in vitro RNA-Seq and explorative proteomic data

We further investigated the overlap between dysregulated pathways in RNA-Seq and proteomic analyses. The proteomically identified pathways matched those found in RNA-Seq data; despite a limited overlap of mRNA and protein regarding significantly affected gene expression products (compare Fig. 2A, C). Comparing the significantly regulated top 20 GO-terms in RNA-Seq with the proteomically identified GO terms, three terms were identical in both datasets. Of note, those pathways were related to ribosomal biogenesis and translation (Fig. 2D). In addition, we analyzed the overlap between KEGG pathways of both transcriptomics and proteomics data and found one identical significantly altered pathway: hsa03010, Ribosome). Importantly, 35% of the compounds of this pathway were significantly dysregulated in one of our screens, indicating an influence of KLF7 on the entire process (*p* < 0.05) (Fig. 2E). Taken together, the GSEA suggested an impact of KLF7 on ribosomal biogenesis.

Specific expression levels of all ribosomal proteins were also analyzed. We identified and quantified 76 ribosomal proteins by mass spectrometry in all samples. Of those, 40 proteins differed significantly between conditions, accounting for 50% of the 60S ribosomal protein subunit and 23% of the 40S subunit (Fig. 2F). Quantification of mRNA of some ribosomal proteins that were dysregulated in proteomics data revealed a significant downregulation of RPL34 and RPL27 in MDA-MB-231 cells (Additional file 1: Fig. S1). This change in protein and mRNA abundance might hint at a specialization of the ribosomes in breast cancer cells in response to KLF7OE.

### Aberrant ribosomal processes detected in breast cancer tissue

We attempted to validate our in vitro findings that KLF7 might regulate ribosomal biogenesis in breast cancer patient samples. Therefore, a publicly available dataset from The Cancer Genome Atlas (TCGA) of 1222 breast cancer patients was stratified according to their KLF7 expression level. GSEA was performed to compare the significantly regulated GO terms in the group with high KLF7 expression and the low expression group (403 vs. 403 samples). Interestingly, among the top six regulated terms were ribosome biogenesis (*p* = 3.4297E−18, *q* = 1.0188E−14), ribonucleoprotein complex biogenesis (*p* = 5.2364E−18, *q* = 1.0374E−14), rRNA metabolic process (*p* = 1.10584E−16, *q* = 1.64244E−13), and rRNA processing (*p* = 2.05484E−16, *q* = 2.03464E−13). In addition, terms related to translation were found, e.g., translation elongation and translation termination with lower significance (Fig. 2G). This confirmed our in vitro data and suggested a role of KLF7 in ribosomal biogenesis in breast cancer tissue.

### Proliferation was unchanged in KLF7 overexpressing cells

**Fig. 3:**
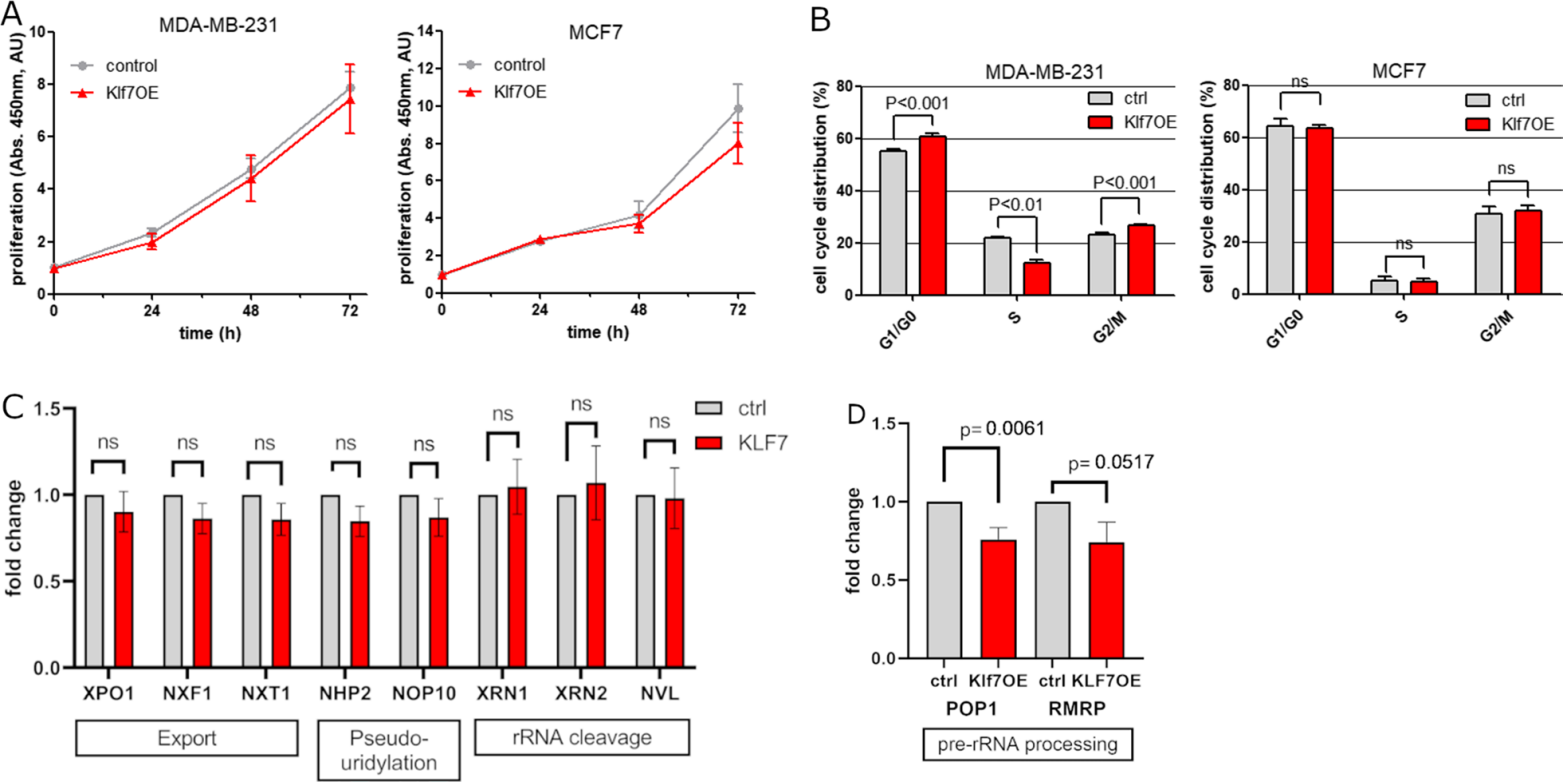
KLF7 expression did not influence proliferation, but nucleolar number and shape. **A** proliferation measured by WST-8 assay in MDA-MB-231 and MCF7 cells showed no change upon overexpression of KLF7 compared to empty vector control. **B** Cell cycle analysis of propidium iodide cells by flow cytometry indicated cell cycle arrest in MDA-MB-231 cells but not in MCF7 cells. **C** mRNA of several genes involved export, pseudouridylation and rRNA cleavage during ribosomal biogenesis in control and KLF7 transfected MDA-MB231 cells demonstrated no change in KLF7OE cells. ns: not significant **D** POP1 and RMRP mRNA involved in pre-rRNA processing were significantly downregulated in KLF7OE cells

Ribosomal production is directly linked to cell growth and proliferation due to the higher need of proteins to be distributed to the daughter cells [47–49]. To investigate whether KLF7 influenced the proliferation of cells, which would in turn explain the identified pathways in ribosome-related processes, we assayed the proliferation of KLF7OE and control cells. Proliferation of MDA-MB-231 and the hormone receptor-positive, luminal type, MCF7 cells overexpressing KLF7 were analyzed using a WST-8 assay. MCF7 cells showed a lower overexpression of KLF7 compared to MDA-MB-231 in qPCR and western blot (Additional file 2: Fig. S2A–D). WST-8 assay revealed no proliferation difference between KLF7OE and control cells during 72 h in both cell types (Fig. 3A). This indicated that the KLF7-regulated alterations in ribosomal processes were a direct effect of KLF7 and not secondary to increased cell divisions. Furthermore, the influence of KLF7-overexpressing cells on cell cycle phases was investigated. We analyzed DNA content of MDA-MB-231 and MCF7 KLF7OE and control cells, which were stained with propidium iodide by flow cytometry. Interestingly, we observed a G1 arrest in KLF7OE conditions in MDA-MB-231. In MCF7 however, no change in cell cycle was observed (Fig. 3B).

### KLF7 might regulate pre-rRNA processing by downregulating POP1 and RMRP

In order to identify pathways that are regulated by KLF7, we screened four processed involved in ribosomal biogenesis for alterations in mRNA levels. mRNAs from export, rRNA cleavage, pseudouridylation and pre-rRNA processing were analyzed by qPCR in MDA-MB-231 and MCF7 cells overexpressing KLF7. Expression levels of members of the export, rRNA cleavage and pseudouridylation processes were not significantly changed (Fig. 3C and Additional file 3: Fig S3A). In contrast POP1 and the lncRNA RMRP which are involved in pre-rRNA processing were significantly downregulated by KLF7OE in MDA-MB-231 cells (Fig. 3D). In the luminal cell line MCF7 no change was observed indicating a different behaviour due to the different molecular subtypes (Additional file 3: Fig S3B).

### KLF7 induced changes in nucleolar number and shape

**Fig. 4:**
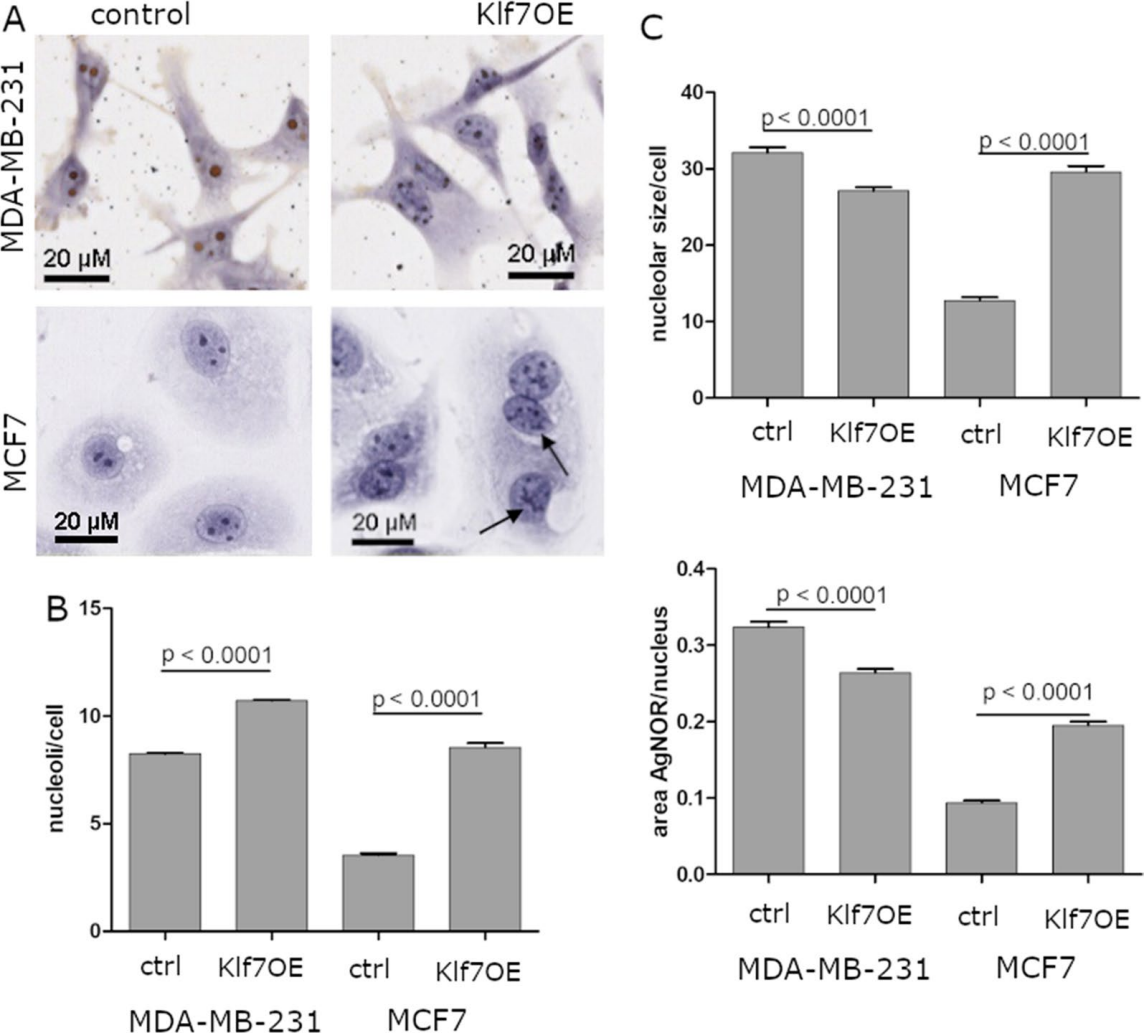
Aberrant nucleoli morphology in vitro. **A** Representative images of AgNOR staining of nucleoli indicated a change in nucleolar morphology (arrows) and number upon KLF7OE in MDA-MB-231 and MCF7 cells. **B** Automatized quantification of nucleolar numbers per cell with QuPath 0.2.3 demonstrated a significant increase in KLF7-overexpressing MDA-MB-231 and MCF7 cells. **C** Nucleolar size per cell and the ratio of AgNOR staining to nuclear area was decreased in MDA-MB-231 cells and increased in MCF7 cells upon KLF7OE

Nucleoli are the sites of ribosomal biogenesis and are formed during pre-RNA processing and subsequent generation of ribosomal subunits. The nucleolar number and morphology, therefore, serves as read-out for ribosomal biogenesis in general [45, 50]. We aimed to identify KLF7-induced alterations in nucleoli in KLF7OE and control cells by AgNOR staining and morphological and quantitative assessment. AgNOR selectively marks the nucleolar organizer regions (NOR) that are associated with ribosomal DNA, and can be considered a marker of rRNA transcriptional activity and ribosomal biogenesis [51, 52]. We observed that KLF7 overexpression in MDA-MB-231 and MCF7 breast cancer cells changed the morphology to more concatenated structures that were comparable to the alterations observed and scored by Stamatopoulou et al. in their iNO score [53] (Fig. 4A). Moreover, the total number of nucleoli per cell increased significantly by 30% and 140% in MDA-MB-231 and MCF7 cells upon KLF7 overexpression (Fig. 4A, B). The size of the nucleoli was also analyzed. In MDA-MB-231 KLF7OE cells, nucleolar size and the ratio of nucleolar to nuclear area significantly decreased by 15% compared to control cells (Fig. 4C). In MCF7 cells, nucleolar size and nucleoli/nuclei ratio significantly increased two to 2.5-fold upon KLF7OE (Fig. 4C). Nuclear size increased by 12% and 7% in KLF7OE in MDA-MB-231 and MCF7 cells, respectively. These results indicated a disruption of nucleolar homeostasis upon KLF7OE, albeit with a different pattern in the triple-negative and hormone receptor-positive cell lines.

### KLF7 increased translation but not transcription

**Fig. 5:**
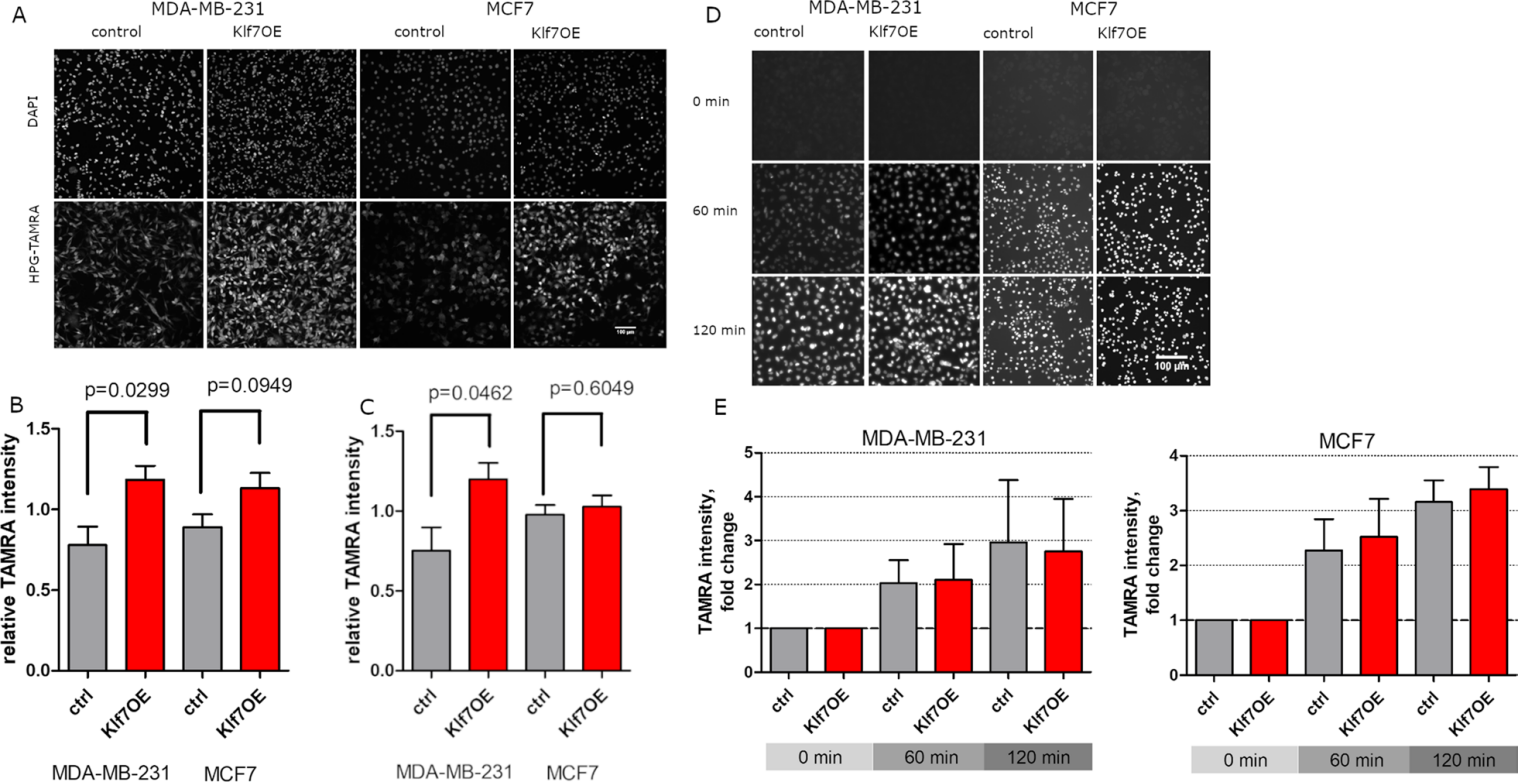
KLF7OE increased transcription but not translation. **A** Fluorescent signal after 45 min of HPG-TAMRA incorporation demonstrated an increased signal in KLF7OE cells. **B** Quantification of the cytoplasmic fluorescent signal showed a significantly increased translation rate in MDA-MB-231 cells and MCF7 cells. **C** Nuclear HPG-TAMRA intensity after 45 min incorporation. **D** Representative images of fluorescent signals after 5-EU incorporation labeling nascent RNA. **E** Cytoplasmic and nuclear intensity of 5-EU-TAMRA was unchanged upon KLF7 overexpression

Ribosomal alterations might lead to changes in overall translation. In our proteomics and transcriptomics GSEA, and in TCGA data stratified according to KLF7 levels, processes related to translation were identified. To detect whether KLF7 influenced translation or transcription, we employed a click-it assay that directly labels newly synthesized protein or mRNA and thereby allows analysis of translational rates [54, 55]. Nascent proteins in MDA-MB-231, and MCF7 control and KLF7OE cells were labeled with L-HPG in methionine-free medium for 45 min and visualized fluorescently with a covalently attached TAMRA molecule using click chemistry. Interestingly, KLF7 expression strongly increased the cells’ global translation, as indicated by increased fluorescence. Overall, cytoplasmic protein expression significantly increased by 50% in MDA-MB-231 cells (*p* = 0.0299) and 27% in MCF7 cells (*p* = 0.0949) (Fig. 5A, B). The nuclear HPG incorporation signal also increased in MDA-MB-231 cells, but not in MCF7 cells, indicating an increase in proteins that were imported in the nucleus (Fig. 5C).

To characterize the effect of KLF7 on transcription, we investigated the overall transcription rate by incorporating 5-EU into nascent RNA over 60 min and 120 min in MDA-MB-231 and MCF7 control and KLF7OE cells. Fluorescent visualization via click chemistry using the same system as for transcription demonstrated no difference in transcription rate between cell conditions (Fig. 5D, E).

### KLF7 expression correlated with tumor grading and molecular subtype in mammary carcinoma

We analyzed the expression of KLF7 in human breast cancer tissue in a TMA cohort of 77 female breast cancer patients, derived from primary biopsies. All patents were chemotherapy naïve. Patients’ age ranged from 30 to 91 years (mean 63 years, standard deviation (SD) 13.57 years). In immunohistochemistry, 60 patients expressed the estrogen- and 50 patients the progesterone-receptor protein. Eleven patients were positive (Score 3) for the receptor tyrosine kinase HER2/neu. According to Goldhirsch et al. [43], 55 patients were classified as Luminal A/B, seven as Luminal Her2, four as Her2 enriched and eleven as triple negative.

**Fig. 6:**
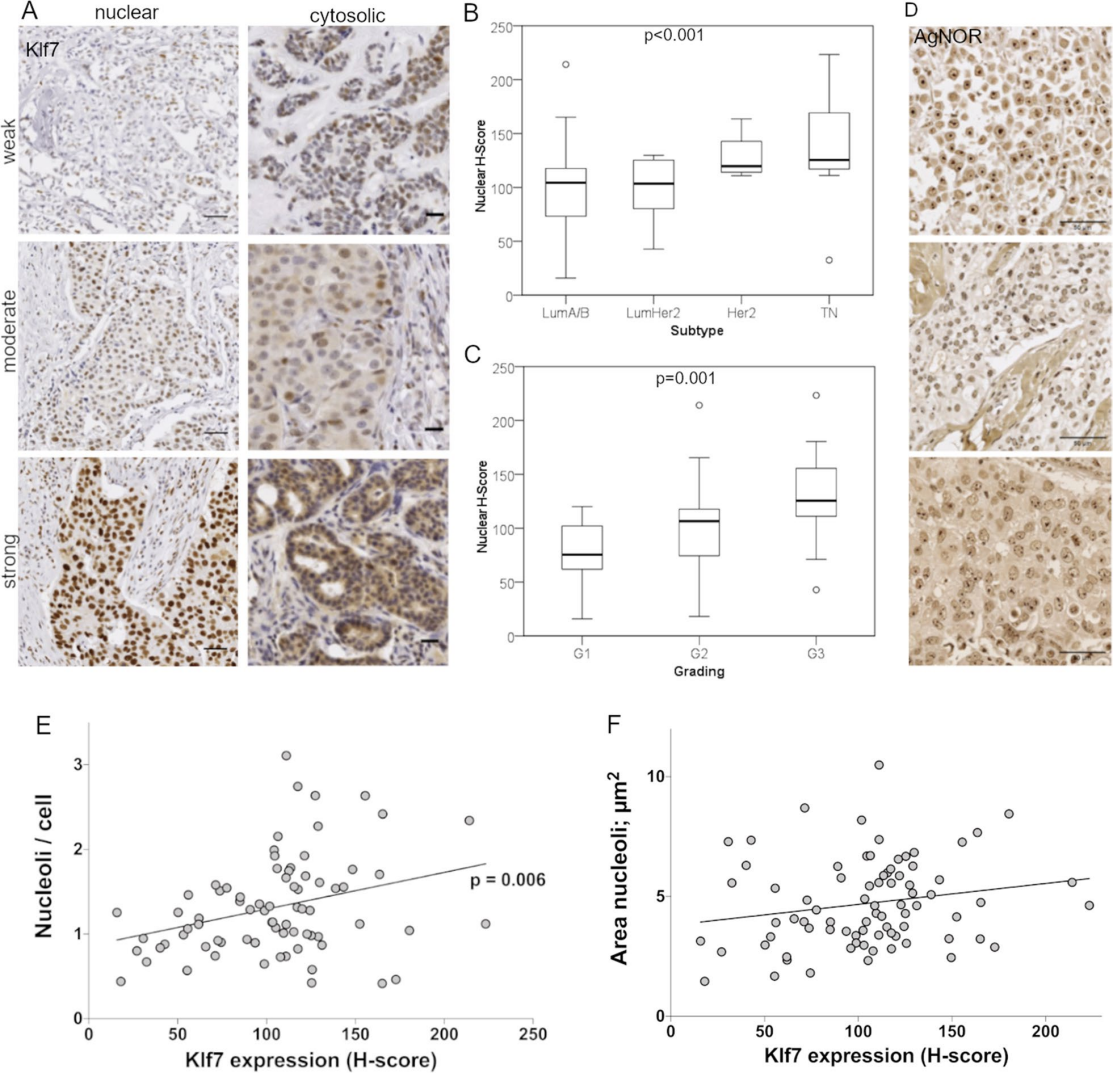
KLF7 correlated with intrinsic subtype, grading, and nucleoli. **A** Representative images of nuclear and cytosolic KLF7 staining in breast cancer tissue samples. Nuclear KLF7 correlated with **B** intrinsic subtype (*p* < 0.001, *n* = 77) and **C** tumor grading (*p* = 0.001, *n* = 77). **D** Representative images of AgNOR staining of nucleoli in breast cancer tissue demonstrated varying nucleolar phenotypes. **E** The number of nucleoli per cell correlated significantly with KLF7 expression, indicated as H-score. **F** The area covered by nucleoli correlated with nuclear KLF7 expression (H-score)

Immunohistochemical KLF7 expression was scored via QuPath 0.2.3 based on the percentage of positive cells and the staining intensity, and expressed as H-score. The nuclear KLF7 signal varied strongly between individuals from strong to absent staining intensity in a range of cells (Fig. 6A). The nuclear KLF7 H-score ranged from 12.75 to 226.43 (SD 40.46). KLF7 expression correlated with clinic-pathological parameters. Nuclear KLF7 expression in breast cancer tissue correlated significantly with the intrinsic subtype (*p* < 0.001) (Fig. 6B) and tumor grading (*p* = 0.001) (Fig. 6C), indicating a role in more aggressive cancer types. Cytoplasmic KLF7 staining has been noticed in breast cancer tissues in the Human Protein Atlas. In addition to the expected nuclear signal, we equally detected cytoplasmic KLF7 staining in some patients (Fig. 6A). The H-score ranged from 1.033 to 176.91 (SD 34.53) (Fig. 6A). H-score of the cytoplasmic KLF7 levels correlated significantly with nuclear KLF7 signal (*p* < 0.001) and intrinsic subtype (*p* = 0.016). No correlations were observed for UICC-, AJCC classification, patients’ age, blood vessel infiltration, lymph vessel infiltration, or perineural invasion.

### KLF7 expression was increased in TP53 mutated patient samples

TP53 is frequently mutated in breast cancer and notably associated mostly with more aggressive tumor types with a poorer prognosis [56]. Given that it has been demonstrated that p53 is a repressor of KLF7 expression in pancreatic cancer [20], we analyzed TP53 and KLF7 in TCGA data. In TCGA breast cancer data, KLF7 expression in tumors holding a functional TP53 mutation was compared against KLF7 in patients with wildtype TP53. KLF7 expression level was significantly higher in TP53-mutated patients (*p* = 0.016). Taken together, our data demonstrated high KLF7 expression levels in aggressive tumor types.

### KLF7 correlated with nucleolar characteristics in breast cancer tissue

To substantiate the role of KLF7 in ribosomal biogenesis in breast cancer, the nucleolar number and size were automatically analyzed in our breast cancer patient cohort via QuPath 0.2.3. Nucleoli were visualized in the tissue biopsies with AgNOR staining. Both the quantity and size of the nucleoli differed strongly between individual patients (Fig. 6D). We correlated nucleolar number and size to nuclear KLF7 expression and identified a significant positive correlation between nuclear KLF7 expression levels (H-score) and the number of nucleoli per cell (*p* = 0.006, Fig. 6E) as well as the nucleolar size (*p* = 0.043, Fig. 6F). AgNOR staining similarly significantly correlated KLF7 expression levels with more aggressive subtypes. Thus, the patient data corroborated our cell culture data and provided a link between KLF7 expression and ribosomal biogenesis in patient tissue.

## Discussion

KLF7—a ubiquitously expressed protein—is strongly expressed in tumor tissues [22]. We aimed to elucidate the molecular function of KLF7 in breast cancer and identified a novel and unexpected role of this protein in ribosomal processes and translation. Regulation of ribosomal biogenesis is one of the most energy-consuming cellular processes and is essential for the adaptation and functioning of cells in physiological and pathological conditions [57]. In comprehensive transcriptomic and proteomic in vitro screens, we identified GO terms indicative of a regulatory role of KLF7 in ribosomal biogenesis. These findings were confirmed by in silico analyses of TCGA breast cancer data showing aberrant cellular processes related to ribosomal biogenesis in high KLF7-expressing breast cancer patients. Consistent with these results, breast cancer tissue and breast cancer cell lines expressing high levels of KLF7 featured disrupted nucleolar morphology and quantity. In vitro, KLF7 overexpression resulted exclusively in increased translation while proliferation and transcription remained unaffected.

To our knowledge, we have found the first connection between a member of the KLF/Sp family of transcription factors and target ribosomal biogenesis processes. This regulation might be important for physiologic and pathophysiologic pathways. So far, the molecular role of KLF7 has mainly been related to proliferation, differentiation, and migration [20, 21, 23–26]. Our results are reminiscent of the wide-ranging importance of the transcription factor MYC, which not only shapes cellular processes such as differentiation, adhesion, or cell cycle through direct transcription and chromatin remodeling but also influences ribosomal biogenesis by regulating the rRNA transcription and ribosomal protein translation [58, 59]. Our data indicate that KLF7 is similarly implicated in ribosomal biogenesis. The main significant GO terms that overlapped in our RNA-Seq and mass spectrometry data were ribosomal biogenesis, mRNA processing, and translation. Those terms were also identified as the most important altered processes related to KLF7 level in patient data from TCGA.

The exact mechanism of ribosomal regulation by KLF7 remains to be investigated. In MDA-MB-231 cells we demonstrated a downregulation RMRP, the RNA component of mitochondrial RNA processing endoribonuclease. RMRP is a non-coding RNA that binds to multiple proteins to form the RNase MRP complex, one of them is POP1 [60]. POP1 has equally been found to be downregulated in MDA-MB-231 KLF7OE cells. The RNase RMP complex is involved in pre-rRNA processing essential for ribosomal biogenesis with an essential role of RMRP [61]. Mutations in RMRP promotor have been correlated with breast cancer [62] and POP1 has been identified as part of a prognostic signature in breast cancer [63]. The changes in POP1 and RMRP expression in MDA-MB-231 indicate a regulatory role of KLF7 in this process in triple-negative cells. The luminal MCF7 cells reacted differently. MCF7 cells are—in contrast to MDA-MB-231—estrogen receptor (ER) positive. In MCF7 cells, ERalpha regulates RMRP which is not the case in ER negative MDA-MB-231 cells [64]. Differences in expression between the MDA-MB-231 and MCF7 cell lines might therefore stem from the molecular subtype of the cell lines which is accompanied with a different tumor biology and patient outcome.

Another potential mechanism is the regulation of ribosomal stoichiometry. The existence of such a specialized cancer ribosome has been detected using methods based on RNA sequencing in human cancer tissue and cell lines [4, 65]. Proteomic analyses in mouse embryonic stem cells also described a heterogeneous ribosomal composition [4]. Interestingly, hereditary diseases that are caused by mutations in ribosomal proteins, the ribosomopathies, increase the risk of developing cancer [66]. KLF7 might therefore play an important role in cancer progression.

Ribosomal biogenesis has been implicated in tumor growth and transformation and has recently been associated with the metastatic capacity of circulating breast cancer tumor cells [5, 17]. In pathological diagnostics, prominent nucleoli as the sites of ribosomal biogenesis have been used for decades to distinguish malignant/tumorous from benign cells [50, 51, 67]. Alterations in nucleolar number or shape have been described for several pathologic conditions [68, 69]. Moreover, ribosomal biogenesis has been suggested as a prognostic marker in breast cancer [70]. We have identified morphological aberrations in nucleoli in breast cancer tissue and cell cultures. In general, more nucleoli were detected in the triple negative cell line MDA-MB-231 compared to the hormone receptor-positive MCF7 cells. This is in line with a recent analysis in breast cancer tissue and cell lines, demonstrating a higher number of nucleoli in TNBC [71]. KLF7 expression significantly increased nucleolar numbers.

We further identified KLF7 as a translation regulator. GO terms related to translation in proteomics and transcriptomics data and our in vitro experiments show that KLF7 increases translation independently of transcription. Moreover, in silico analysis of human tissue samples demonstrated that GO terms related to translation are significantly altered depending on KLF7 levels. Dysregulated translation efficiency represents a hallmark of cancer [72]. Translation is not only related to proliferation but also to the cancer cell plasticity driven by stressors like hypoxia and energy deprivation [73]. This change in translation is often decoupled from transcription but is controlled at the level of initiation, elongation, termination and protein folding – processes co-determined by the ribosome. As has been reported for Akt and Ras signaling in gliomas, KLF7 upregulates translation independently of increased transcription rate. In Ras/Akt signalling, specific cellular processes such as growth, transcription, and morphology regulation are most likely to be affected by this effect [74]. Supposably, the effect of KLF7 might likewise apply to a subset of pathways, e.g., cytoplasmic processes such as cell substrate junctions or vesicle trafficking that we have identified by RNA-Seq. How these processes are regulated by KLF7 remains to be investigated. Ribosomal translation might be altered by changes in ribosomal stoichiometry. In bacteria, differing ribosomal composition leads to distinct translational profiles [75]. Similar effects have been observed in eukaryotes, with important implications for pathologic conditions [65, 76]. This is in line with the observation that ectopic expression of RPL15 increases overall expression in breast-circulating tumor cells [17].

We further identified KLF7 as an important contributor to the aggressiveness of breast cancer. Nuclear KLF7 expression levels correlated significantly with the breast cancer subtypes and grading. Higher-graded tumors and more aggressive intrinsic tumor types, namely triple-negative breast cancer, showed high expression levels of KLF7. Triple-negative breast cancer is also more frequently associated with mutated TP53 and a dismal prognosis for the patients [56]. In TP53-mutated tumors with higher p53 expression level, an increased area covered by nucleoli has been described and AgNOR staining correlated with tumor size and grading [77]. Our data indicate that TP53 influenced KLF7 expression level. We speculate that in TP53-mutated cancer types, KLF7 levels are increased leading to alterations in ribosomal processes and more aggressive progression.

KLF7 was detected not only in the nuclei in breast cancer tissue but also in the cytoplasm, which corroborates observations of the Human Protein Atlas. Cytoplasmic KLF7 expression correlated with the intrinsic subtype and nuclear staining. A role of KLF7 in Golgi apparatus has been recently demonstrated in pancreatic cancer [20]]. We speculate that the nuclear and cytoplasmic KLF7 expression might be involved in different cellular processes; the cytoplasmic KLF7 proteins could be involved in affecting the Golgi apparatus, while nuclear KLF7 could contribute to ribosomal regulation.

## Conclusion

In conclusion, we identified a novel link between ribosomal biogenesis and KLF7 in mammary carcinoma. By qualitatively and quantitatively influencing the cellular proteome, KLF7 expression in cancer cells might have far reaching implications for the tumor biology and the aggressiveness of the cancer.

## Supplementary Information

**Additional file 1: Figure S1.** Expression of mRNA of ribosomal proteins in MDA-MB-231 cells. qPCR of the depicted genes in KLF7 transfected MDA-MB-231 cells reveal downregulation of RPL34 and RPL27 mRNA.

**Additional file 2: Figure S2.** KLF7 expression in MCF7 cells. A. mRNA in KLF7 transfected MCF7 cells was strongly enhanced compared to control cells B. western blot of MCF7 protein lysate demonstrated increased protein level in KLF7OE cells. C. Densitometric quantification of western blots showed significantly higher KLF7 levels (n = 4). D. Nuclear localization of KLF7 in MCF7 cell spheres.

**Additional file 3: Figure S3.** Analysis of processes involved in ribosomal biogenesis in MCF7 cells. A) KLF7OE does not lead to significant changes in mRNA expression of the indicated genes in export, pseudouridylation, rRNA cleavage. B) No changes in POP1 and RMRP mRNA level by KLF7OE in MCF7 cells.

## Data Availability

Proteomic, transcriptomic and patient datasets analysed during the current study are available from the corresponding author on reasonable request. The transcriptomics datasets analysed during the current study are available in the TCGA repository.

## Abbreviations

KLF7: Krüppel-like factor 7
TNBC: Triple negative breast cancer
RNA-Seq: RNA sequencing (RPS9, RPS14, RPL5, RPL10, RPL11, and RPL39)
GSEA: Gene set enrichment analysis
KLF7OE: KLF7 over expression
TCGA: The Cancer Genome Atlas
FFPE: Formalin fixed, paraffin embedded
GO: Gene ontology
NOR: Nucleolar organizer regions
5-EU: 5-Ethynyl uridine
L-HPG: L-homopropargylglycine
