# Supplementary figures and images for: Krüppel-like factor 7 influences translation and pathways involved in ribosomal biogenesis in breast cancer
Source: Breast Cancer Res. 2022 Oct 3;24:65. doi: 10.1186/s13058-022-01562-8 (PMC9531505; doi:10.1186/s13058-022-01562-8)

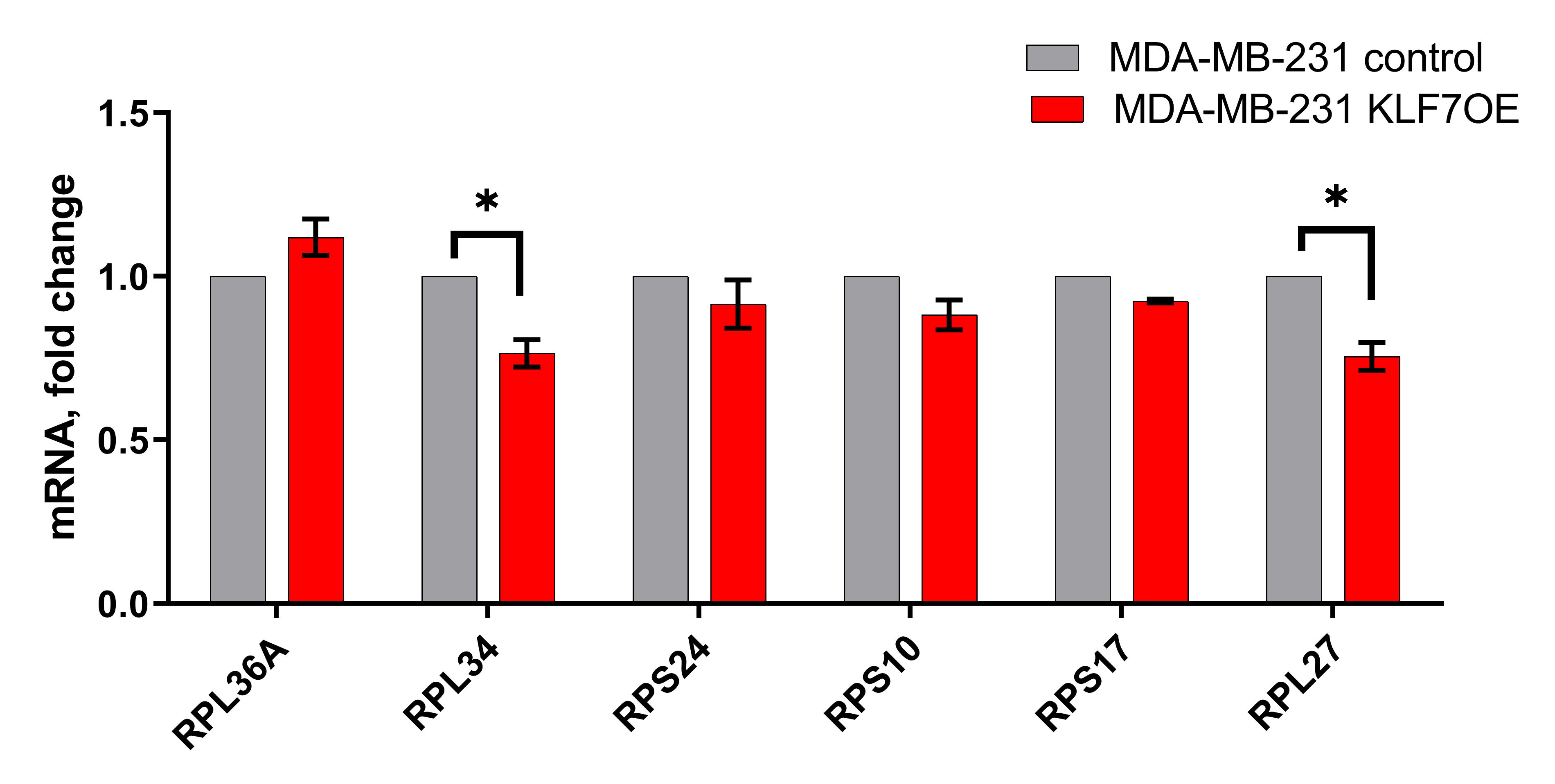

Supplement: Supplementary file 1 — Additional file 1: Figure S1. Expression of mRNA of ribosomal proteins in MDA-MB-231 cells. qPCR of the depicted genes in KLF7 transfected MDA-MB-231 cells reveal downregulation of RPL34 and RPL27 mRNA. [file 13058_2022_1562_MOESM1_ESM.tiff]

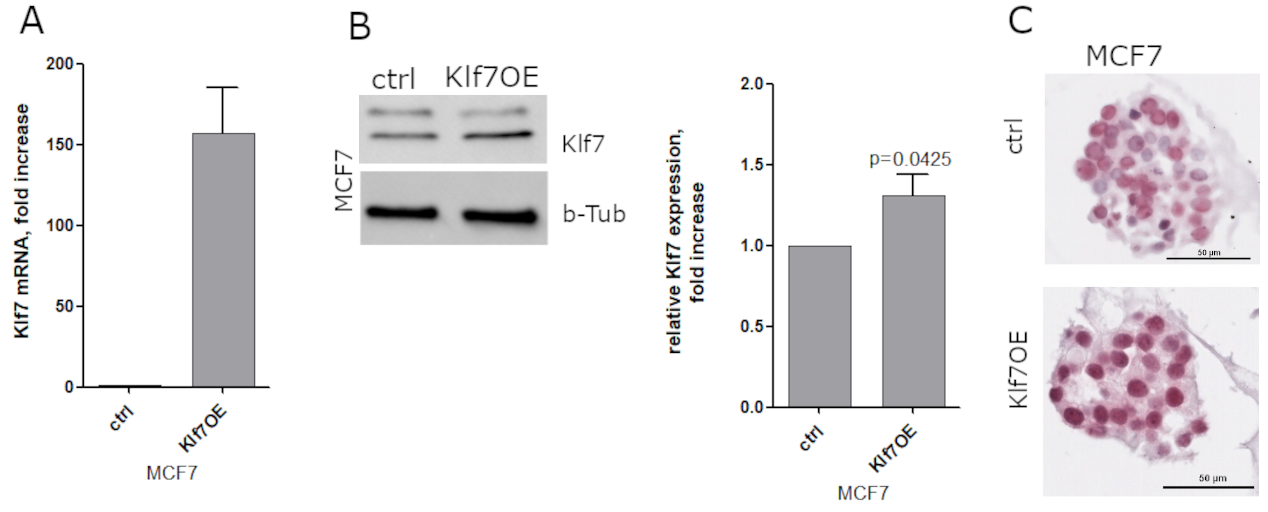

Supplement: Supplementary file 2 — Additional file 2: Figure S2. KLF7 expression in MCF7 cells. A. mRNA in KLF7 transfected MCF7 cells was strongly enhanced compared to control cells B. western blot of MCF7 protein lysate demonstrated increased protein level in KLF7OE cells. C. Densitometric quantification of western blots showed significantly higher KLF7 levels (n = 4). D. Nuclear localization of KLF7 in MCF7 cell spheres. [file 13058_2022_1562_MOESM2_ESM.tiff]

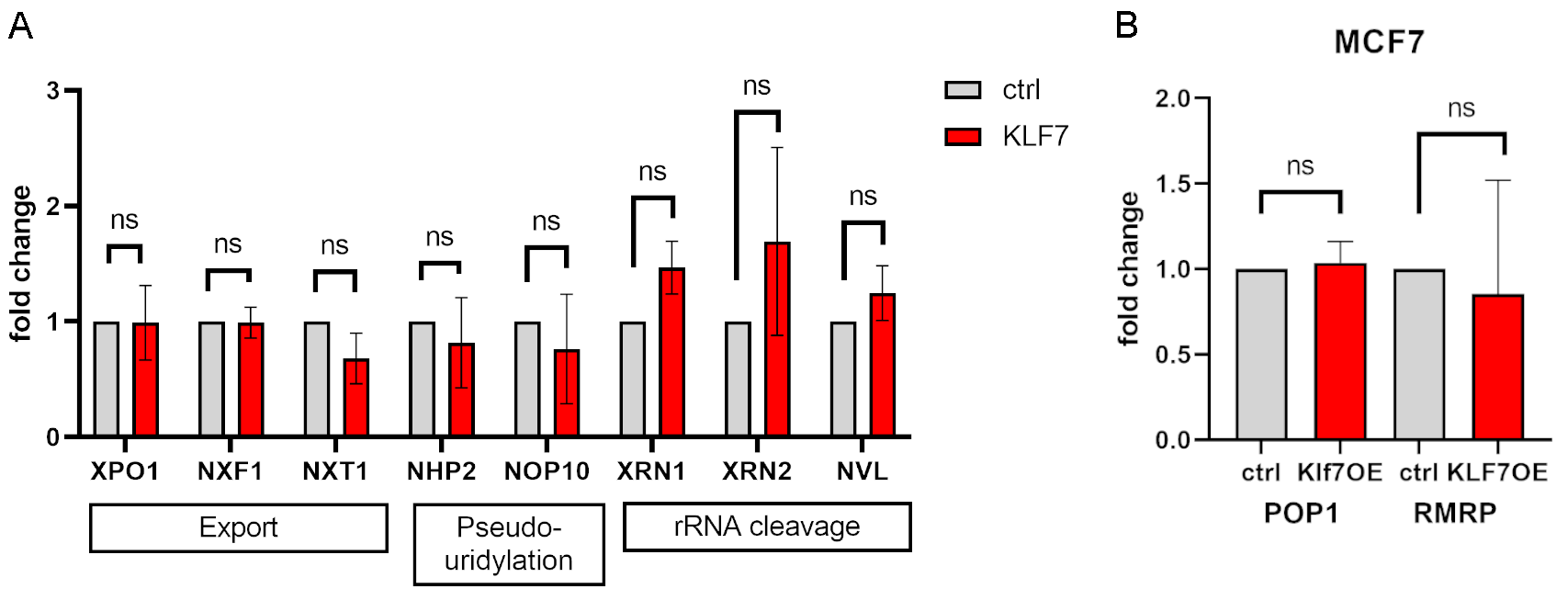

Supplement: Supplementary file 3 — Additional file 3: Figure S3. Analysis of processes involved in ribosomal biogenesis in MCF7 cells. A) KLF7OE does not lead to significant changes in mRNA expression of the indicated genes in export, pseudouridylation, rRNA cleavage. B) No changes in POP1 and RMRP mRNA level by KLF7OE in MCF7 cells. [file 13058_2022_1562_MOESM3_ESM.tiff]
